# Interaction between isoprene and ozone fluxes in a poplar plantation and its impact on air quality at the European level

**DOI:** 10.1038/srep32676

**Published:** 2016-09-12

**Authors:** Terenzio Zenone, Carlijn Hendriks, Federico Brilli, Erik Fransen, Beniamio Gioli, Miguel Portillo-Estrada, Martijn Schaap, Reinhart Ceulemans

**Affiliations:** 1 Department of Biology, Centre of Excellence on Plant and Vegetation Ecology (PLECO), University of Antwerp, B-2610 Wilrijk, Belgium; 2 TNO, Department of Climate, Air and Sustainability, P.O. Box 80015, 3508 TA, Utrecht, the Netherlands; 3 National Research Council, Institute of Agro-Environmental and Forest Biology (IBAF-CNR), Via Salaria Km 29,300 – 00016 Monterotondo Scalo, Roma, Italy; 4 National Research Council, Institute for Sustainable Plant Protection (IPSP-CNR), Via Madonna del piano 10, 50017, Sesto Fiorentino, Italy; 5 StatUa Centre for Statistics, University of Antwerp, Prinsstraat 13, B-2000 Antwerp, Belgium; 6 National Research Council, Institute of Biometeorology (IBIMET-CNR), Via G. Caproni 8, 50145, Firenze, Italy

## Abstract

The emission of isoprene and other biogenic volatile organic compounds from vegetation plays an important role in tropospheric ozone (O_3_) formation. The potentially large expansion of isoprene emitting species (e.g., poplars) for bioenergy production might, therefore, impact tropospheric O_3_ formation. Using the eddy covariance technique we have simultaneously measured fluxes isoprene, O_3_ and of CO_2_ from a poplar (*Populus*) plantation grown for bioenergy production. We used the chemistry transport model LOTOS-EUROS to scale-up the isoprene emissions associated with the existing poplar plantations in Europe, and we assessed the impact of isoprene fluxes on ground level O_3_ concentrations. Our findings suggest that isoprene emissions from existing poplar-for-bioenergy plantations do not significantly affect the ground level of O_3_ concentration. Indeed the overall land in Europe covered with poplar plantations has not significantly changed over the last two decades despite policy incentives to produce bioenergy crops. The current surface area of isoprene emitting poplars-for-bioenergy remains too limited to significantly enhance O_3_ concentrations and thus to be considered a potential threat for air quality and human health.

Isoprenoids represent an important and abundant class of biogenic volatile organic compounds (BVOCs); isoprene (C_5_H_8_, 2-methyl-1,3,-butadiene) comprises about half of the total BVOCs emitted globally^1^. Reactive isoprenoids in the atmosphere, and in particular isoprene, feed the cycle NO–NO_2_–O_3_ that is responsible for the formation^2^ and degradation^3^ of tropospheric ozone (O_3_). A recent study^4^ showed a carbon assimilation reduction of up to 19% in *Pinus ponderosa* and *Citrus* plantations, as a result of deposition of O_3_ to the canopy, while the estimated yield losses caused by elevated ground-level [O_3_] ranged between 7% and 16% for agricultural crops^5^. Moreover, a modeling study^6^ has predicted that isoprene emissions would increase in Europe from 11.5 Tg C yr^−1^ to 16.0 Tg C yr^−1^ as a consequence of an assumed expansion of bioenergy crops, as poplar (*Populus*), with subsequent important increases in ground-level [O_3_], affecting crop yields and human health. Poplars are well known to be strong emitters of isoprene^7^,^8^ and the emissions are highly light and temperature dependent^9^,^10^. Isoprene emissions increase with temperature up to 40 °C^10^, even when carbon assimilation is declining^11^. This uncoupling of isoprene emission from the photosynthetic process reinforces the theory that isoprene may protect plants against heat stress^12^,^13^. The response of isoprene emission to increasing [O_3_] has primarily been examined in laboratory experiments under controlled environments (i.e. by employing enclosures) that might not reflect real-life conditions. Moreover in most of these studies^4^,^14^,^15^,^16^ high concentrations of O_3_ (100–300 ppb) were applied for a short term (i.e., days to weeks) to simulate stress. In particular, no long-term observations of isoprene emissions and O_3_ fluxes under uncontrolled field conditions have been made so far. We therefore run simultaneous ecosystem-level measurements of isoprene and O_3_ fluxes by using the eddy covariance technique and investigated the influence of [O_3_] on both isoprene emission and total O_3_ uptake.

In addition, we have scaled-up the measured isoprene emissions through the chemistry transport model (CTM) LOTOS-EUROS associated with the current surface area planted with poplar-for-bioenergy in Europe, as reported by the Food and Agriculture Organization of the United Nations^17^, and by the European Biomass Association^18^. This allowed us to quantify the potential impact of the isoprene fluxes on ground-level [O_3_].

## Results

### Experimental observations

**Figure 1:**
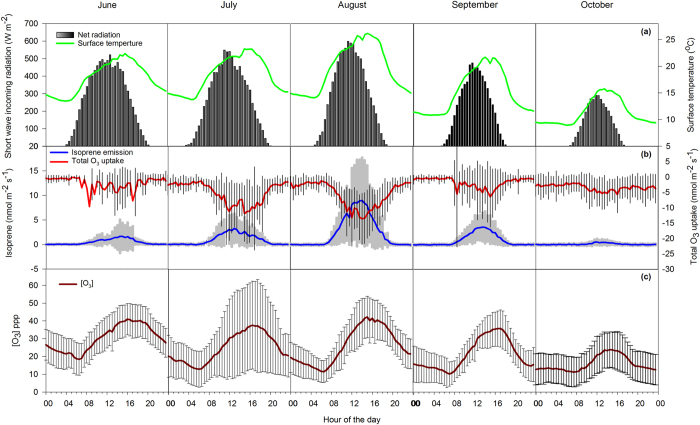
Diurnal cycles of (**a**) net radiation and surface temperature; (**b**) total O_3_ uptake and isoprene fluxes and (**c**) [O_3_] concentrations. The diurnal cycles of fluxes represent averages calculated from the monthly half-hour eddy covariance data during the months June-October 2012.

**Figure 2:**
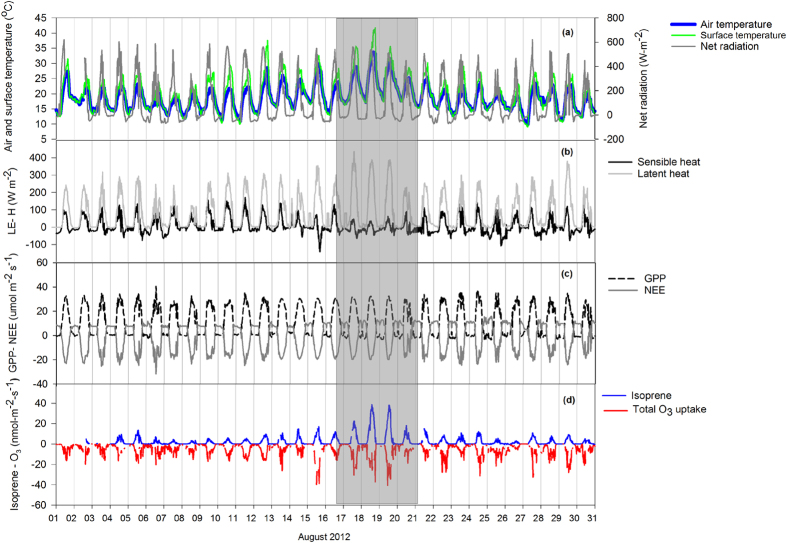
Average daily variation measured during August 2012 of (**a**) net radiation, air temperature and surface temperature; (**b**) energy exchange as latent heat (*LE*) and sensible heat (*H*); (**c**) CO_2_ flux as net ecosystem exchange (NEE) and gross primary production (GPP); and (**d**) isoprene emission and total O_3_ uptake. The shaded area represents the days characterized by a peak of isoprene emission.

Measured isoprene fluxes showed a well-defined daily cycle with maximum emission rates during the afternoon, from midday till 16.00 CEST (Fig. 1b), and corresponding with maximum values of incoming short-wave radiation (Rg) (Fig. 1a), while surface temperature (*S*_t_) peaked about four hours later (Fig. 1a). Total O_3_ uptake (Fig. 1b) also showed a diurnal trend that mirrored the isoprene fluxes and also occurred in parallel with increasing [O_3_] (Fig. 1c). Uptake of O_3_ was dominated by the stomatal component (Supplementary information Fig. S3) that represented 70% of the total O_3_ uptake. When looking at the entire season, isoprene fluxes were characterized by a remarkable peak emission occurring for a few days in August with the highest values reaching 38.6 nmol m^−2^ s^−1^ on 18 August 2012 and 38.0 nmol m^−2^ s^−1^ on 19 August 2012 (Fig. 2d). During these days (Fig. 2, shaded area), an increment of about 10 °C (around midday) in both S_t_ and *Air*_t_ (Fig. 2a), an increase in the fluxes of latent heat (*LE*) (Fig. 2b) and of stomatal O_3_ uptake (Fig. 2d), as well as a simultaneous decrease in the fluxes of sensible heat (*H*) (Fig. 2b) were observed. Despite these changes, Rg (Fig. 2a), gross primary production (GPP) and net ecosystem exchange (NEE) (Fig. 2c) did not show any significant change during the month of August 2012.

The Pearson correlations were computed between isoprene fluxes, O_3_ uptake, [O_3_], Rg, radiation and *Air*_t_.(Table 1). The largest correlation for O_3_ uptake was observed with [O_3_] (*r* = 0.49). The effects of [O_3_], Rg, and *Air*_t_ on either O_3_ uptake or isoprene fluxes were assessed using general linear models (GLM), revealing that [O_3_], Rg and *Air*_t_ had a highly significant effect on both O_3_ uptake and isoprene fluxes. Based upon the GLM, we calculated the fraction of the variance of either isoprene fluxes or O_3_ uptake attributable to [O_3_], Rg and *Air*_t_, respectively (Table 2). A GLM that considers only Rg and *Air*_t_ explained 16.6% of the variance of O_3_ uptake. Adding [O_3_] to the same model increased the *r*^2^ from 0.166 to 0.287. Furthermore, a GLM that takes into account only [O_3_] explained 24.4% of the variance in O_3_ uptake. As for isoprene fluxes, when the GLM included both Rg and *Air*_t_, it was able to explain 54% of the variance. In particular, by adding [O_3_] to the same GLM (that already included both Rg and *Air*_t_), the variance explained for isoprene fluxes increased by just 1% (from 54 to 55%). On the other hand, a GLM that considered only [O_3_] explained 21% of the variance in isoprene fluxes.

### Model simulations

Simulated isoprene and O_3_ fluxes were validated against the observations by running the LOTOS-EUROS model in which the vegetation of the entire 0.125 × 0.0625 degree grid cell, where the experimental site is located, was set to poplar plantation (run ‘Basic-zoom’). Since in our study case the resulting footprint for the flux measurements was entirely covered by poplars, this approach gave a fair comparison between simulated and measured fluxes of O_3_ and isoprene.

The comparison between observed and simulated [O_3_] as well as the comparison between observed and simulated O_3_ uptake showed a reasonable agreement (SI Fig. S1). However, fast fluctuations in O_3_ fluxes (especially deposition peaks) were not captured well by the LOTUS-EUROS model as the underestimation of O_3_ fluxes mainly occurred at times when the O_3_ fluxes were very large for a short while. On average, LOTOS-EUROS overestimated the O_3_ flux by 22%. The comparison between modeled and observed isoprene fluxes (SI Fig. S4) showed that the model underestimated the seasonal variability of isoprene emissions in late summer (August and September) whereas the observed emissions were overestimated in June. A comparison between simulated and observed [O_3_] from 47 rural sites belonging to the European Monitoring and Evaluation Programme EMEP (SI Fig. S2) showed an average model bias of 2.7 ppb with an average hourly temporal coefficient of determination (*r*^2^) of 0.59 throughout all the stations. When model simulations of the [O_3_] daily maxima were compared to the observations, a better agreement with an average bias of 0.67 ppb and an *r*^2^ of 0.76: the slopes of both plots (0.74 and 0.77 for average and daily maximum respectively) indicate that LOTOS-EUROS underestimates the spatial variability and daily maximum [O_3_] (SI Fig. S2).

**Figure 3:**
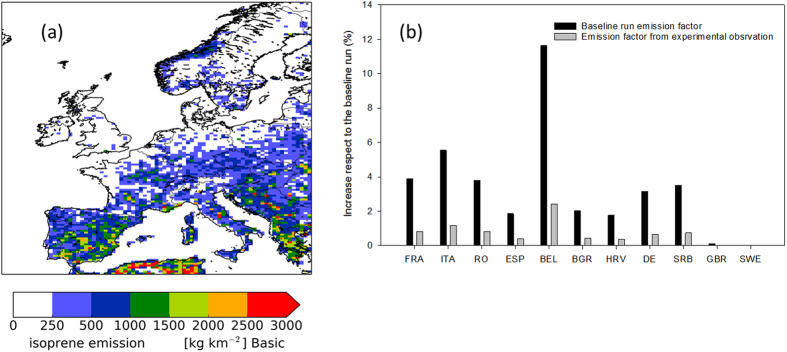
Isoprene emissions in Europe simulated by the LOTOS-EUROS model: (**a**) isoprene emission distribution for the basic run; (**b**) increase in isoprene emissions (in %) for an increased land surface area of poplar plantations and using emission extracted from measurements of this study (poplar-emisfac). The map was generated using the LOTOS-EUROS model version 1.10.005. URL link: www.lotos-euros.nl.

Isoprene fluxes obtained using the basic configuration of the LOTOS-EUROS chemistry transport model with a resolution of 0.5 × 0.25 degrees and the land use database CORINE 2006 (ID run named “basic” in SI Table 2) showed that the largest emissions were associated with isoprene emitting forests (Fig. 3a). The north-south gradient in isoprene emission rates reflected the temperature gradient across Europe, as southern Mediterranean areas feature the highest temperatures with respect to Northern ones. After incorporating into the LOTOS- EUROS model the areas covered by poplar plantations as reported by the FAO for the different countries in Europe (Table S3) and selecting the vegetation-specific isoprene emission factor for poplar plants (ID run “poplar” in SI Table 2) isoprene fluxes increased from 0.01% to 11.6% compared to the “basic” run. However, when a vegetation-specific isoprene emission factor obtained from the present experimental observations was applied to LOTUS-EUROS (ID run named “poplar emission factor” in SI Table 2), total isoprene emissions were 26% lower compared to the LOTOS-EUROS basic configuration and a maximum increase of 2.4% of the isoprene emissions was obtained (Fig. 3b). Overall, the simulated isoprene fluxes increased from 3.4 to 3.5 Tg C for the period of April-October 2012 when the present surface area of poplar plantations was included in LOTOS-EUROS model by using the vegetation-specific isoprene emission factor obtained in our observations.

**Figure 4:**
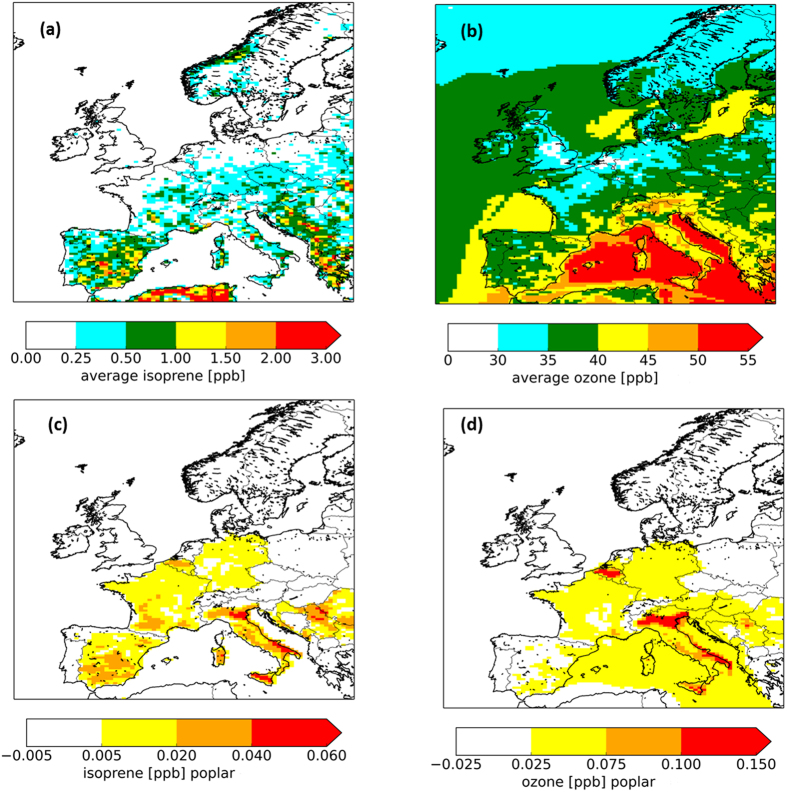
Average concentrations for the period April - September 2012 as simulated by the LOTOS-EUROS model, basic run for isoprene (**a**) ozone (**b**); and the difference (in ppb) caused by an increase in poplar plantation area for isoprene (**c**) and [O_3_] (**d**). The map was generated using the LOTOS-EUROS model version 1.10.005. URL link: www.lotos-euros.nl.

In this study, the isoprene concentration pattern (Fig. 4a) mapped the actual distribution of isoprene emissions in Europe: indeed very low concentrations (<1 ppb) were displayed in north-western Europe whereas in southern Europe concentrations reached 2–3 ppb. Differently from isoprene concentrations, [O_3_] (Fig. 4b) ranged between 20 and 50 ppb for central Europe. The lowest levels of [O_3_] are found in regions with high NO_x_ levels such as central England, the Ruhr area in Germany, the Netherlands and the Paris region in France, where ozone titration during the night plays an important role. [O_3_] generally increase towards southern Europe, with the highest values found along the Mediterranean coast. When poplar plantations were included in the land-use map, an increase in [O_3_] ranging from 0.025 to 0.15 ppb (Fig. 4d) was observed in areas where poplar plantations were located, caused by an increase of isoprene concentrations from 0.02 to 0.06 ppb (Fig. 4c). The increase in [O_3_] highlighted in Fig. 4c represents the net impact of the isoprene fluxes emitted by poplar plantations: overall [O_3_] increased by 0.1–0.5% while O_3_ deposition in poplars increased by 0.1–0.3%.

## Discussion

The highest isoprene fluxes observed in this study occurred in mid-August 2012 (Fig. 1), when isoprene synthase activity was maximized by the increase of the air and surface temperatures under high light intensities in a fully developed canopy where strong isoprene emitting adult leaves outweighed the young ones^19^. As the poplars were not water stressed^20^ during the whole growing season, they kept their stomata open and increased the fluxes of evapotranspiration in an attempt to reduce the leaf temperature, although the cooling effect due to isoprene emission was negligible (as it accounted for only 0.1–0.3% of the cooling effect of water). Because the amount of Rg did not change (during the measurements period), fluxes of *H* decreased simultaneously to increased LE, causing the observed reduction of the Bowen ratio. However, GPP and NEE were not affected by this thermal variation; therefore poplar plants emitted isoprene at high rates in the field when the photosynthetic machinery resulted undamaged by stress. This evidence further suggests that isoprene makes the chloroplasts membranes more resistant and more performing under high temperatures as observed in the past^12^, also in laboratory trials^21^. Indeed, experiments employing *Populus* grown in pots^22^ have evidenced the role of isoprene in unstressed plants, as the chloroplasts of isoprene emitting plants dissipated less energy as heat than the chloroplasts of non-emitting plants, when exposed to physiologically high temperatures (29–38 °C) that did not damage the photosynthetic apparatus. While the process of isoprene emission is crucial for tropospheric O_3_ formation in the atmosphere, the [O_3_] itself also has important consequences on the physiology of plants by creating a feedback loop that influences the production and emission of isoprene^4^. Nevertheless, the response of isoprene emission to [O_3_] may vary considerably and is related to the level and the duration of the O_3_ exposure. It has been hypothesized^4^,^23^ that the response of isoprene emission to increasing doses of [O_3_] follows the most common form of the hormetic dose–response curve. The effect of moderate [O_3_] on isoprene emission has been investigated in *Populus nigra* plants in a laboratory experiment^15^, which showed that the emission of isoprene and oxygenated six-carbon (C6) volatiles were inhibited when plants were exposed to an [O_3_] of 80 ppb for two weeks. However, the impact of [O_3_] on isoprene emission has been investigated in several other laboratory studies with conflicting results: some studies highlighted that high doses of O_3_ enhanced isoprene emission^15^,^23^,^24^, whereas others^25^,^26^,^27^ reported an inhibition of isoprene emission in poplars under more usual low doses of [O_3_].

The experimental observations of isoprene and O_3_ fluxes were used, through the application of the LOTOS-EUROS model, to investigate to what extent existing poplar plantations influence ground level [O_3_] and found their impact to be small. Our results show that isoprene fluxes affected the average [O_3_] during the period April-September (2012) by max. 0.5%, thus indicating that the poplar plantations currently in place for biomass production do not significantly alter the [O_3_]. The slight increase in [O_3_] was also partially counteracted by the increased O_3_ deposition; poplars are characterized by high LAI and are effective O_3_ sinks^28^.

The maximum effect of isoprene emission on [O_3_] was calculated using the highest emission factor (60 μg gDM^−1^ hr^−1^) regarding isoprene emission from poplar trees reported in the literature, where it was found to range between 45 and 60 μg gDM^−1^ hr^−1^ 18,29,30. However, both the isoprene emission factor and leafy biomass derived from our experimental observations provides a 26% lower total isoprene emission than that resulted from LOTOS-EUROS model in the basic configuration. A misrepresentation of the seasonal variability of isoprene emissions in LOTOS-EUROS caused an underestimation of observed isoprene fluxes in September. This bias influenced O_3_ production in the model, but since peaks of [O_3_] mainly occured before September 2012, the impact on our conclusions is expected to be small. The observations presented here are representative for the northern temperate climate zone of Europe, which contains the countries with the highest area of poplar plantations (SI Table 3). Since the relationship used in LOTOS-EUROS model to simulate isoprene emissions from poplar plantations on the basis of the observations is temperature-dependent^30^, LOTUS-EUROS produced reliable results for the rest of Europe. On the other hand, the isoprene emission factor representing the agricultural land employed here to run the LOTOS-EUROS model is lower than that used by both Ashworth *et al.*^31^ and Lathière *et al.*^32^. This means that the impact of poplar plantations on the [O_3_] simulated in our study may be relatively high considering that isoprene emission factor and leafy biomass quantities of poplars used in LOTOS-EUROS are on the high end of the values reported in literature. However, total isoprene emission resulting from our study was lower than that reported in e.g. Ashworth *et al.*^6^ which accounted for 11.5 Tg C yr^−1^ while in Beltman *et al.*^33^ it was estimated to be 5 Tg C for the period April-September (2012). The differences in the estimates of isoprene emission between past studies and our present work are caused by differences in domain (in Ashworth *et al.*^6^ plantations expanded much further east) and by a diverse time period of analysis (only summertime has been considered in Beltman *et al.*^33^, whereas the whole year in Ashworth *et al.*^6^). So far, simulations of isoprene fluxes from poplar plantations have mainly used future scenarios^31^,^32^,^33^, where the impacts of isoprene emissions on [O_3_] levels were driven by the assumption of a very large increment in both the area of poplar plantations and in the extent of the emission factor chosen to perform the simulations. Therefore, results from past simulations suggested that isoprene fluxes emitted from the foreseen increasing biomass production in expanding poplar-for-bioenergy plantations could potentially increase the risk of O_3_ damage to human health, crops and ecosystems. As a consequence, to counteract the increasing level of tropospheric [O_3_] might require a large investment for the abatement of NO_x_^33^. The impact of domestic biomass production for fuel and power generation needed to meet EU targets for 2020 regarding the [O_3_] is small, but significant (Ashworth *et al.*^6^). In particular, the study of Ashworth *et al.* (2012)^6^ assumed that 72 Mha of poplar were to be planted to meet the projected use of biomass as a renewable energy source, leading to a 39% increase in isoprene emissions. Moreover other authors^34^ suggested that, especially in eastern Europe, a large proportion of agricultural land might have been converted into biomass forest.

However, despite policies encouraging the use of biomass for energy are already in place^35^, data from the literature confirmed the trend that the area covered by poplar plantations did not show any significant changes over the last 20 years, at least in the countries considered in our study (ref. 17, Table S3).The current area of poplar SRC plantations present in Europe (Table S4) is far too small to create a threat for O_3_ formation, leading to only 1% extra isoprene emissions, as demonstrated by our simulation. If anthropogenic emissions of non-methane volatile organic compounds (NMVOC) are underestimated in our model study, which is not unlikely as these emissions are quite uncertain, the impact of isoprene emissions on O_3_ formation calculated here could be overestimated. Hence, our study indicates that increasing the production of bioenergy from poplar plantations in Europe would not reduce the air quality in the short-term; therefore policymakers should not be concerned in supporting policies that encourage the planting of bioenergy crops.

## Material and Methods

The research site was a poplar (*Populus*) bioenergy plantation located in Lochristi, East-Flanders (Belgium; 51°06′44″N, 3°51′02″E) at an elevation of 6 m above sea level. The plantation was established in April 2010 with 12 selected clones of *Populus deltoides*, *P. maximowiczii, P. nigra,* and *P. trichocarpa*, at a density of 8000 plants ha^−1^ on a surface of 18.4 ha. The main environmental conditions and stand characteristics are reported in Table S1. The research site was equipped with two different eddy covariance (EC) systems. One system was used to monitor the CO_2_, latent heat (*LE*), sensible heat (*H*) and O_3_ fluxes between the ecosystem and the atmosphere and comprised a three-dimensional sonic anemometer (model CSAT3, Campbell Scientific, Logan, UT, USA) to measure the wind speed components, a fast response LOZ-3F O_3_ analyzer (Drummond Technology Inc., Ontario, Canada) and a fast response CO_2_/H_2_O infrared analyzer (LI-7000, LI-COR, Lincoln, NE, USA). The LOZ-3F O_3_ analyzer is based on chemi-luminescence with Eosin-Y dye circulated continuously through a peristaltic pump in the sample cell, and it measures the O_3_ mixing ratio at a 10 Hz sampling frequency. A slow response O_3_ analyzer (API 400E, Teledyne Instruments, CA, USA) was used to continuously monitor [O_3_], which was then compared with the concentrations measured by the LOZ-3F, to determine whether any drifting had occurred in the signal of the LOZ-3F. The API instrument was calibrated every six months by the Flemish Environment Agency (www.vmm.be). The second EC system monitored the fluxes of biogenic volatile organic compounds (BVOCs) and included a sonic anemometer (model USA1, Metek GmbH, Elmshorn, Germany) coupled with a proton transfer reaction “Time-of-Flight” mass spectrometer (PTR–TOF–MS) (Ionicon, Innsbruck, Austria) that measured the volume mixing ratios (VMRs) of BVOCs. The data streams of the anemometer and the PTR–TOF–MS were acquired independently by two different computers and synchronized with dedicated software (NTP, Network Time Protocol, University of Delaware, DE, USA) to an independent external clock through the internet, with an accuracy of<20 ms. The two EC systems were completely independent, although they were installed very close to each other with a 1 m spatial separation between the inlets of the two sampling lines.

Fluxes of O_3_ were calculated using EddyPro (version 5.2.0): the mean lateral and vertical velocity components were forced to zero by a two-component rotation. The maximum cross covariance function (within the 30-min averaging time) was used to determine the lag time for O_3_, which was about 2 s. A frequency response correction was performed according to Moncrieff *et al.*^36^.

The fluxes of CO_2_ and of water vapor were calculated using the EdiRe software (R. Clement, University of Edinburgh, UK; www.geos.ed.ac.uk/abs/research/micromet/EdiRe/).

In order to reduce the burden of PTR-TOF-MS data analysis, the overall raw dataset collected during the measuring campaign was post-processed by the routine programs of Müller *et al.*^37^ to screen for the presence of emitted/deposited fluxes of the most common protonated ions unambiguously found to be related to a BVOC. After post-processing, PTR-TOF-MS data were background corrected by subtracting ambient VOC-free air generated by a gas calibration unit (GCU) (Ionimed, Innsbruck, Austria) and calibrated regularly with the same gas standard (Apel Riemer, USA) during the length of the field campaign to quantify the VMRs of the selected BVOCs. This latter technique has been previously described in detail^11^. In order to standardize the computation of isoprene fluxes with the EC method the EddyPro software was modified into a new customized version that was named EddyVoc. Similarly to EddyPro, the processing routine programmed in EddyVoc masked the raw data with a quality flag to exclude individual spikes, values out-of-range and background calibration periods of the PTR-TOF-MS. Further details about the EC data processing were provided previously (Brilli *et al.*^11^).

### Estimation of stomatal O_3_ fluxes

Stomatal resistance to water vapor (*R*_sto_, Eq. 1) was calculated from the EC measured evapotranspiration using the evaporative/resistance method^38^:


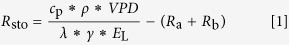


where *λ* is the latent heat of vaporization in air (J kg^−1^), *γ* is the psychrometric constant (0.065 kPa K^−1^), *E*_L_ is the transpiration rate (kg H_2_O m^−2^ s^−1^) after subtracting the evaporative contribution from the soil, *c*_p_ is the specific heat of air (J kg^−1^ K^−1^), *ρ* (kg m^−3^) is the density of dry air, *VPD* is the vapor pressure deficit at leaf level (kPa), *R*_a_ and *R*_b_ (s m^−1^) are the aerodynamic, respectively, laminar sublayer resistances. Stomatal conductance to ozone was calculated as the inverse of *R*_sto_ corrected for the difference in diffusivity between O_3_ and water vapor. Stomatal O_3_ flux was calculated by multiplying the stomatal conductance to O_3_ by [O_3_] assuming that inter-cellular [O_3_] was zero.

### Statistical analysis

To investigate the influence of [O_3_] on the isoprene emission and the total O_3_ uptake we used a correlation and a regression model approach. Due to the skewed distribution of the O_3_ uptake and isoprene emissions, these variables were logarithmically transformed to be used as outcome variable in the regression analysis. Since the logarithms of a negative number cannot be calculated, we calculated the log(-O_3_ uptake+1) and log(isoprene emission + 3) respectively. These log-transformed values were used to calculate Pearson correlation (r) coefficients. For the multivariable linear regression models [O_3_], Rg, and *Air*_t_ were entered as independent variables. The log-transformed values of either isoprene emission or O_3_ uptake was entered as dependent variable.

Calculation of the partial *r*^2^ for [O_3_] in the model with either isoprene emission or O_3_ uptake, was carried out comparing the *R*^2^ between a regression model with [O_3_], Rg, and *Air*_t_ and a regression model with only Rg, and *Air*_t_. The difference in *r*^2^ between these two models represents the fraction of the variance in the outcome (either isoprene emission or O_3_ uptake) that can only be attributed to [O_3_]. Linear regression models were fitted using the lm() function in the statistical software package R, version 3.1.2.

### The CTM LOTOS-EUROS model

The LOTOS-EUROS v1.10 is a 3-D regional chemistry transport model that simulates air pollution in the lower troposphere. For a more detailed description of the model reference is made to Schaap *et al.*^39^. The model uses a normal longitude–latitude projection and allows the user to specify the resolution and domain within its master domain that encompasses Europe and its periphery. For this work, runs were performed at a 0.5 × 0.25 degree resolution for the European domain (15° E – 35° W, 30–60° N) and at 1/8 × 1/16 degree for a domain containing Belgium. The model ceiling is at 3.5 km above sea level and consists of three dynamical layers: a mixing layer and two reservoir layers above. The height of the mixing layer at each time and position is extracted from the ECMWF meteorological data used to drive the model. The height of the reservoir layers is set to the difference between the ceiling and mixing layer height. Both layers are equally thick with a minimum of 50 m. If the mixing layer is near or above 3500 m high, the top of the model exceeds 3500 m. A surface layer with a fixed depth of 25 m is included in the model to monitor ground-level concentrations.

Advection in all directions is handled with the monotonic advection scheme developed by Walcek^40^. Isoprene chemistry and hydrolysis of N_2_O_5_ were described by Adelman^41^ and by Schaap *et al.*^42^, respectively. Stomatal resistance is described by the parameterization of Emberson *et al.*^43^ and the aerodynamic resistance is calculated for all land-use types separately. Isoprene emissions were calculated based on detailed information about tree types in Europe: a land use dataset^44^ was combined with the distributions of 115 tree species over Europe^29^. During each simulation time step, isoprene emissions, for each grid cell, were calculated as a function of the biomass density and standard emission factor of the species or land-use class, taking into account the growing season of deciduous trees and agricultural crops. Moreover, the role of the local temperature and photosynthetically active radiation were taken into account in the biogenic emissions by following the empirically designed algorithms described by Guenther *et al.*^30^. Anthropogenic emissions were taken from the TNO-MACC database^45^ which is a widely used emission database for air quality modelling. Emission totals and trends reported in the TNO-MACC database are in line with what is reported in other emission databases for Europe^46^,^47^. The model set-up used here did not contain secondary organic aerosol formation or a volatility basis set approach. The different model runs were analyzed and compared in terms of isoprene emissions, isoprene and O_3_ concentrations. Modeled O_3_ concentrations were validated using measured concentrations from the European Monitoring and Evaluation Programme (EMEP) monitoring network (www.emep.int). Only background stations situated in rural areas below 700 m height were considered.

## Additional Information

**How to cite this article**: Zenone, T. *et al.* Interaction between isoprene and ozone fluxes in a poplar plantation and its impact on air quality at the European level. *Sci. Rep.* **6**, 32676; doi: 10.1038/srep32676 (2016).

## Supplementary Material

Supplementary Information

## Figures and Tables

**Table 1:** Pearson correlation coefficients of the investigated variables.

Variable	Pearson correlation (*r*)				
	log(isoprene emission	log(O_3_ uptake)	[O_3_]	Rg	Air_t_
log(isoprene emission	1	0.45	0.46	0.59	0.65
log(O_3_ uptake)	0.45	1	0.49	0.32	0.36
[O_3_]	0.46	0.49	1	0.34	0.55
Rg	0.59	0.32	0.34	1	0.45
Air_t_	0.65	0.36	0.55	0.45	1

For isoprene emission and ozone uptake Pearson correlations of the log-transformed values were calculated since these variables were highly non-normal. This log transformation reversed the sign of the correlations for O_3_ uptake. log(isoprene emission) = logarithm of isoprene emission (nmol m^−2^ s^−1^). log(O_3_ uptake) = logarithm of O_3_ uptake (nmol m^−2^ s^−1^). O_3_ uptake = total ozone uptake (nmol m^−2^ s^−1^). [O_3_] = ozone concentration (ppb). Rg = short-wave incoming radiation (W m^−2^). Air_t_ = air temperature (°C).

**Table 2:** Correlation coefficients (*r* ^2^ values) for the GLM with the log(-O_3_ uptake) and log(isoprene Fc) as dependent variables, and [O_3_], short-wave incoming radiation, (Rg) and air temperature (*Air*_t_) as independent variables.

Model outcome	Variable	*r*^2^
log(-O_3_ uptake)	Air_t_ + Rg + [O_3_]	0.28
	Air_t_ + Rg	0.16
	Difference by [O_3_]	0.12*
	[O_3_]	0.24
log (isoprene Fc)	Air_t_ + Rg + [O_3_]	0.55
	Air_t_ + Rg	0.54
	Difference by [O_3_]	0.01*
	[O_3_]	0.21

^*^Partial r^2^ gained by including [O3] in the model.
