# Supplementary material for: Interaction between isoprene and ozone fluxes in a poplar plantation and its impact on air quality at the European level
Source: Sci Rep. 2016 Sep 12;6:32676. doi: 10.1038/srep32676 (PMC5018846; doi:10.1038/srep32676)
Supplement: Supplementary Information [file srep32676-s1.doc]

Supplemental Information Appendix

Interaction between isoprene and ozone fluxes in a poplar plantation and its impact on air quality at the European level

Terenzio Zenone^a^, Carlijn Hendriks^b^, Federico Brilli^c,d^, Erik Fransen^f^, Beniamio Gioli^e^, Miguel Portillo-Estrada^a^, Martijn Schaap^b^, and Reinhart Ceulemans^a^

^a^ Department of Biology, Centre of Excellence on Plant and Vegetation Ecology (PLECO), University of Antwerp, B-2610 Wilrijk, Belgium.

^b^ TNO, Department of Climate, Air and Sustainability, P.O. Box 80015, 3508 TA,

Utrecht, the Netherlands.

^c^ National Research Council, Institute of Agro-Environmental and Forest Biology (IBAF-CNR), Via Salaria Km 29,300 – 00016 Monterotondo Scalo, Roma, Italy.

^d^ National Research Council, Institute for Sustainable Plant Protection (IPSP-CNR), Via Madonna del piano 10, 50017, Sesto Fiorentino, Italy.

^e^ National Research Council, Biometeorology Institute (IBIMET-CNR), Via G. Caproni 8, 50145, Firenze, Italy.

^f^ StatUa Centre for Statistics, University of Antwerp, Prinsstraat 13, B-2000 Antwerp, Belgium

Corresponding author: Dr. Terenzio Zenone.

University of Antwerp. Department of Biology, Centre of Excellence on Plant and Vegetation Ecology (PLECO), Universiteitsplein 1, B-2610 Wilrijk, Belgium

E-mail: [Terenzio.Zenone@uantwerpen.be](mailto:Terenzio.Zenone@uantwerpen.be)

Phone + (32) 3-265 2831; Fax: + (32) 3-265 2271.

Table1. Main environmental and site characteristics of the site investigated: air temperature (in °C), photosynthetically active radiation (PAR; in µmol m^-2^ s^-1^), stomatal conductance (*G*_sto_; in mol m^-2^ s^-1^), soil water content (SWC; in %), evapotranspiration (ET; in mm month^-1^), rainfall (in mm month^-1^), leaf area index (LAI; in m^2^ m^-2^) canopy height (in m).

*n*.*a*.= not assessed

|  | Air temperature | | | PAR | | | *G*_sto_ | | | SWC | | | ET | Rainfall | LAI | Canopy height |
| --- | --- | --- | --- | --- | --- | --- | --- | --- | --- | --- | --- | --- | --- | --- | --- | --- |
|  | average | min | max | average | min | max | average | min | max | average | min | max | Monthly sum | Monthly sum |  |  |
| June | 17.46 | 4.64 | 32.67 | 714.40 | 58.86 | 1531 | 0.41 | 0.08 | 1.42 | 0.30 | 0.19 | 0.36 | 61.3 | 91.0 | 0.93 | 0.87 |
| July | 18.31 | 8.07 | 38.03 | 781.00 | 72.40 | 1492 | 0.45 | 0.02 | 1.64 | 0.34 | 0.21 | 0.42 | 67.2 | 145.0 | 2.90 | 1.72 |
| August | 19.67 | 9.06 | 41.74 | 782.55 | 142.17 | 1375 | 0.44 | 0.06 | 1.54 | 0.21 | 0.12 | 0.31 | 93.5 | 40.2 | 4.62 | 2.76 |
| September | 15.19 | 34.5 | 4.73 | 638.93 | 64.41 | 1224 | 0.47 | 0.04 | 2.06 | 0.14 | 0.12 | 0.18 | 68.4 | 13.0 | 4.59 | 3.55 |
| October | 13.28 | 0.98 | 26.22 | 494.89 | 39.02 | 944 | 0.49 | 0.07 | 1.94 | 0.19 | 0.15 | 0.30 | 28.1 | 98.2 | 2.82 | 3.73 |
| Average | 16.7 | 11.4 | 28.6 | 682.3 | 75.3 | 1313 | 0.45 | 0.05 | 1.72 | 0.23 | 0.15 | 0.31 | *n.a.* | *n.a.* | *n.a.* | *n.a.* |
| Total | *n.a.* | *n.a.* | *n.a.* | *n.a.* | *n.a.* | *n.a.* | *n.a.* | *n.a.* | *n.a.* | *n.a.* | *n.a.* | *n.a.* | 318.5 | 387.4 | *n.a* | *n.a.* |

Table 2. Overview of model runs with the LOTOS-EUROS chemistry transport model: CORINE2006 = land use database. CORINE2006 + FAO poplar data = CORINE2006 land use database, with the area of poplar plantations reported by FAO for the country listed in Table S3 .

| **Run-ID** | **Land use** | **Source of emission factor isoprene SRC poplar** | **Resolution** |
| --- | --- | --- | --- |
| Basic | CORINE2006 | Koeble and Seufert 2001 (48) | 0.5 x 0.25 deg |
| Basic-zoom | CORINE2006 | Koeble and Seufert 2001 (48) | 0.125 x 0.0625 deg |
| Poplar | CORINE2006 + FAO poplar data | Koeble and Seufert 2001(48) | 0.5 x 0.25 deg |
| Poplar-emis_fact | CORINE2006 + FAO poplar data | Measurements presented in this paper | 0.5 x 0.25 deg |

Table 3. Land surface areas (in 10^3^ ha) of planted poplar in Europe. Source: FAO 2012 (37)

|  | 1992 | 1996 | 2000 | 2004 | 2008 | 2012 |
| --- | --- | --- | --- | --- | --- | --- |
| Country |  |  |  |  |  |  |
| France | 279 | 245 | 236 | 236 | 236 | 236 |
| Hungary | 150 | 162 | 157 | n.a | n.a | n.a |
| Turkey* | 130 | 150 | 145 | 130 | 125 | 125 |
| Italy | 79 | 71 | 119 | 118 | 118.5 | 118 |
| Romania* | 83 | 83 | 113 | n.a | 55.3 | 55 |
| Spain | 91 | 98 | 103 | 66.7 | 98.5 | 98 |
| Belgium | n.a | 45 | 40 | 35 | 32.5 | 32 |
| Bulgaria | n.a | n.a | 25.6 | 20.6 | 18.9 | 20 |
| Croatia | n.a | n.a | 19.5 | 13.2 | 12 | 12 |
| Germany | n.a | 103 | 103 | 100 | 100 | 100 |
| Serbia & Monten.* | n.a. | n.a. | n.a. | 35.9 | 33.1 | 33 |
| UK | n.a. | n.a. | n.a. | 1.3 | 1.3 | 1.3 |
| Sweden | n.a. | n.a. | 0.2 | 0.2 | 0.3 | 0.3 |
| Netherland | n.a. | 31 | n.a. | n.a | n.a. | n.a. |
| Total | 812 | 988 | 1061.3 | 756.9 | 831.4 | 830.6 |

* Country not EU member

Table 4. Land surface areas (in 10^3^ ha) of planted poplar short rotation coppice (SRC) culture in Europe (2015). Source: Association Europeen de de la Biomasse (AEBIOM;50)

| Country | Areas |
| --- | --- |
| Austria | 1 |
| Denmark | 6 |
| France | 2.5 |
| Germany | 6.8 |
| Hungary | 4 |
| Ireland | 3 |
| Italy | 6.5 |
| Poland | 6 |
| Sweden* | 12 |
| UK | 2.5 |
| Total | 50.3 |

*Willow plantations

Figure S1 Comparison between eddy covariance (EC) flux measurements and model simulations of the hourly data of (a) O_3_ concentrations, and (b) O_3_ fluxes.


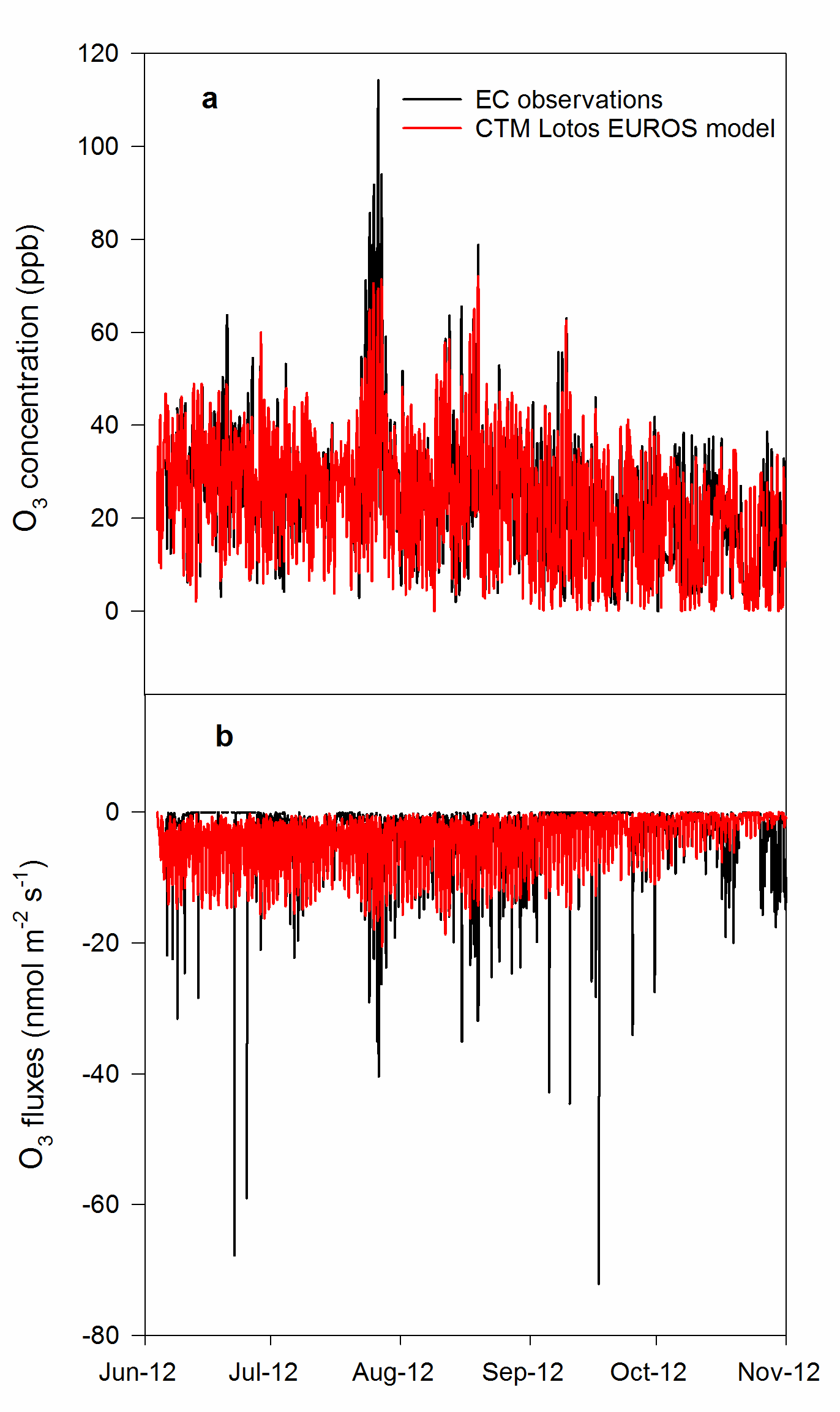


Figure S2. Observed vs. modeled average ground level O_3_ concentrations (a) and average daily maximum concentrations (b) during April-September 2012 for the European Monitoring and Evaluation Programme (EMEP) rural background stations.

 
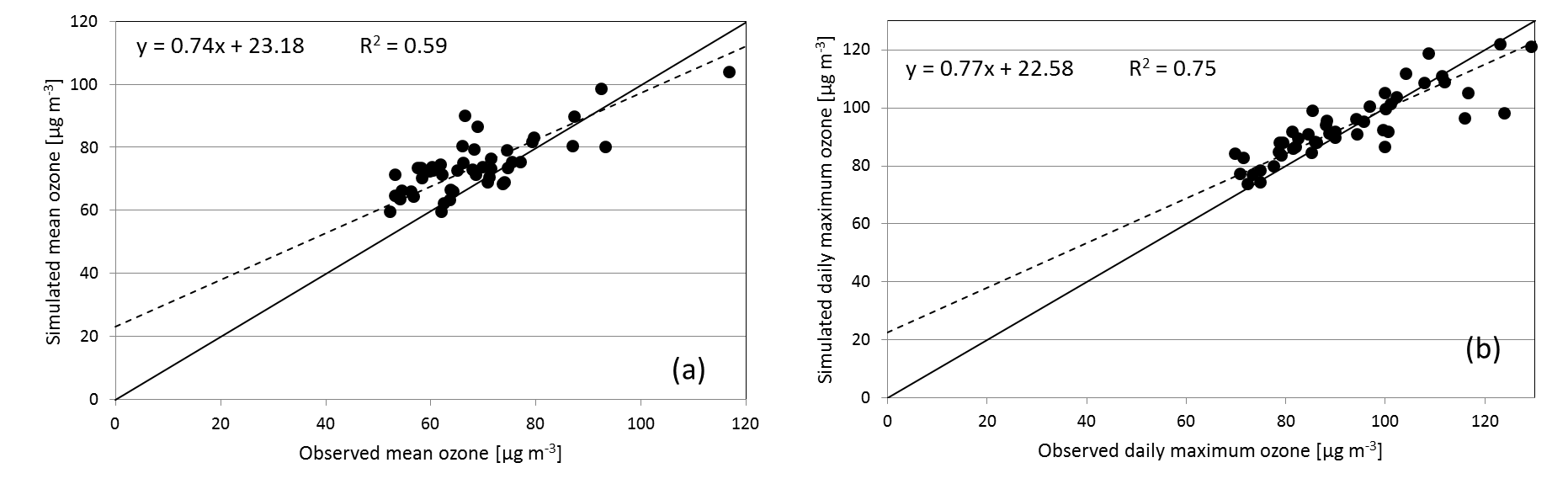


Figure S3. Thirty-minute average diurnal cycles (± standard deviation) of total and stomatal O_3_ uptakes measured during the 2012 growing season
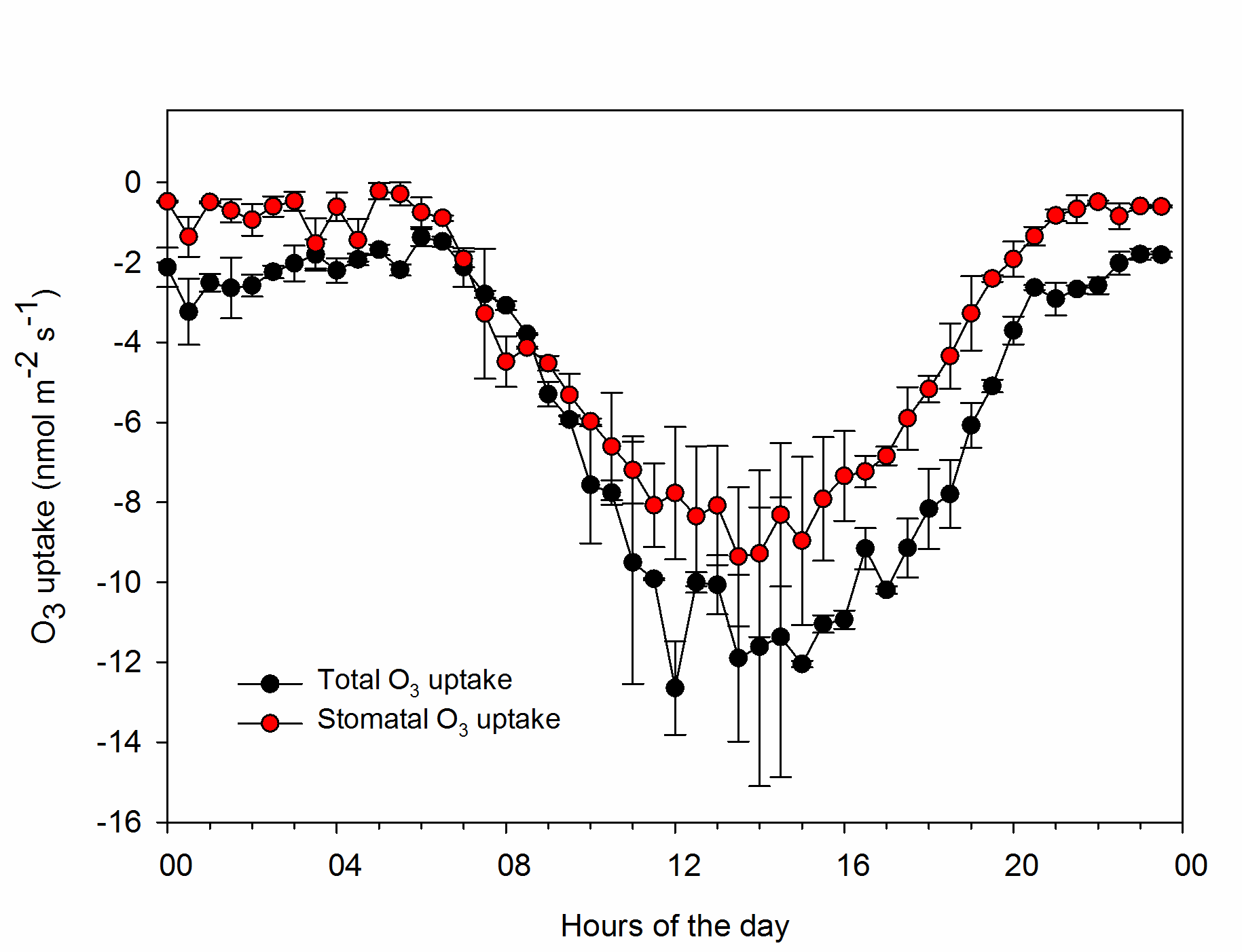


Figure S4. Comparison between observed and modeled isoprene fluxes during August (panel A; *r*^2^ 0.37), July (panel B; *r*^2^ 0.33), June (panel C; *r*^2^ 0.14) and September (panel D; *r*^2^ 0.02)
